# Infiltrating the Curriculum: Integrating Medical History with Existing Surgical Pathology Tutorials

**DOI:** 10.15694/mep.2020.000044.1

**Published:** 2020-03-12

**Authors:** Joshua Teo, Aram Cox, Tiffany Ngu, Huan Doan, Sankar Sinha

**Affiliations:** 1 University of Tasmania

**Keywords:** infiltrating medical curriculum, medial history, medical education, peer to peer teaching, history of medicine

## Abstract

This article was migrated. The article was marked as recommended.

**Objective:** To assess medical students’ perspective on medical history embedded into a pre-existing learning module.

**Design, Setting, and Participants:** This study was performed in 2018 at the University of Tasmania; participants were medical students in year three of the Bachelor of Medicine Bachelor of Surgery course. This was a cross-sectional study which used a mixed-method survey before and after a lecture series to assess medical students’ perspectives on history of medicine.

**Intervention:** Historical perspectives were incorporated into existing surgical pathology tutorials.

**Main outcome measures:** Students completed a survey on medical history before and after the lecture series. The survey used both qualitative and quantitative measures to assess students’ perception of the utility of medical history and how it was taught in this project.

**Results:** In the initialquestionnaire, students indicated they believed medical history would help make them better doctors and enhance their learning of pathology. In the final questionnaire, students agreed that learning medical history was important in becoming a doctor. Students enjoyed the content and found the integration of history and pathology beneficial to learning.

**Conclusion:** This study demonstrates one method by which to increase medial history teaching without major alterations to an existing medical curriculum.

## Introduction

### Does knowledge of medical history produce better doctors?

Currently, there is no research-based evidence on this; Duffin (1995, p159) writes “..that a person can be an excellent physician with little or no knowledge of medical history, but the same is probably true for certain basic science subjects, such as biochemistry and molecular genetics”. On the contrary, Hippocrates reported: “The physician should know what the physician before him has known if he does not want to deprive himself and others” ( Metcalf & Stuart, 2014). Clearly, there are arguments for and against the inclusion of medical history as part of the medical curriculum. In Australia, most medical schools are of the view that history is not an essential component of physician training ( Wijesinha & Dammery, 2008). Additionally, history of medicine is not mentioned in the Australian Medical Council standards for Assessment and Accreditation of medical schools ( Australian Medical Council, 2012).

Huynh from the University of New South Wales in Australia states “It is surprising that it (history of medicine) is not taught more often in medical school.” ( Huynh, 2017, p673). Medical schools have undertaken various methods by which to include more history in their respective curriculums,highlighting the numerous barriers to incorporating history in medical education ( Lederer, 1995; Shedlock, 2012). These include limited resources, curricular time and skepticism.

This article describes a project aiming to provide students at the University of Tasmania with formal medical history teaching embedded in their routine learning activities.

The research questions were:

1. Is it feasible to deliver elements of history of medicine within an existing curriculum?
2. Does this format enhance student engagement and interest in learning medicine?

## Methods

This study was undertaken during the 2018 academic year within the Bachelor of Medicine Bachelor of Surgery (MBBS) Course at the University of Tasmania, Australia (UTAS). This is a five-year undergraduate programme with roughly 120 students enrolled in each cohort. Students in year three undertake a surgical rotation, which includes a blended learning program in surgical pathology delivered over four sessions. Topics included various aspects of general surgery, including upper gastrointestinal tract, hepatobiliary system, lower gastrointestinal tract, and breast and thyroid.

For each session, digital images of surgical pathology specimens from the UTAS pathology museum were presented online with an attached discussion board. One week prior to a two-hour face-to-face (f2f) tutorial, students used the discussion board to identify and answer questions regarding the pathology specimens. Discussion included how the pathology might have manifested in patients, along with the evolution of management over time. Responses were then collated and discussed during the f2f tutorial session.

For this project, 15 minutes of each f2f session were dedicated to delivering historical perspectives relevant to the topic. Historical perspectives focused on eponyms and the evolution of surgical management; these were prepared and presented by final year medical students. Sessions were repeated four times during the course of the academic year involving roughly 120 (30 X 4) students. Historical perspectives used in the tutorials included Marshall and Warren’s discovery of Helicobacter pylori ( Prabhu & Shivani, 2014), the controversy surrounding the naming of Crohn disease ( Smith & Wakefield, 1994), Meckel and his encounter with Napoleon Bonaparte ( Haubrich, 1998), the romantic story behind Halsted’s introduction of rubber surgical gloves, and his unfortunate addiction to cocaine ( Rankin, 2006).

### Assessment and evaluation of the program

Prior to the first session, details and aims of the project were explained to students. It was explained that choosing to participate would not have a direct positive or negative impact on academic achievement. All data collected would be deidentified immediately, and would only be shared amongst those analysing the data. Students who chose to participate signed a consent form. Those who chose not to participate were still invited to attend the tutorials.

An initial survey was conducted prior to the first f2f session, followed by a second survey after the last f2f session. Both surveys consisted of scaled response (Likert scale) and open-ended questions. Scaled response answers ranged from one, indicating strong disagreement with the statement, to five indicating strong agreement. The open response questions evaluated the anticipated and actual positive/negative consequences of the project.

The initial survey gauged students’ perception on the impact that medical history would have on their future practice, and the quality of medical history teaching students received during their first two years of medical school. The final survey focused on what students thought about the lecture series after its completion and whether they viewed it as an effective method of teaching history. The survey questions were based on those used at the University of Liverpool (U.K.) in their development of a medical history course ( Sheard, 2006).

After each lecture, a formative quiz was administered which focused on historical perspectives in order to consolidate students’ learning. Results from these quizzes were not included in this study.

### Ethics

Ethics approval was obtained from the Tasmanian Social Sciences Human Research Ethics Committee (Ref: H0017066).

## Results/Analysis

### Initial Questionnaire

**Figure 1.**
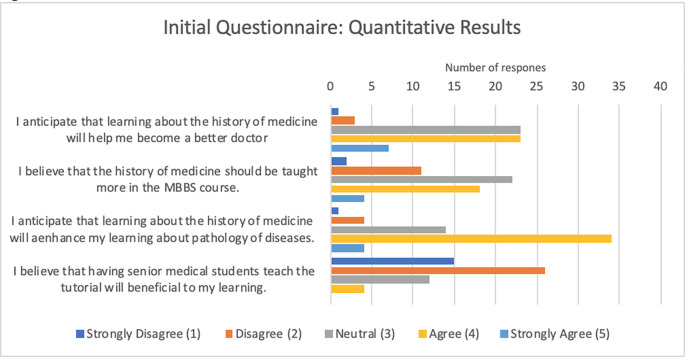
Initial Questionnaire: Quantitative Results

On the initial questionnaire, students indicated that they believe learning history would help make them better doctors (3.56/5.00) and that it would enhance their learning of pathology (3.63/5.00). Students also indicated that history should be taught more in the medical curriculum (3.19/5.00). Furthermore, students disagreed with the idea that having senior medical students teach would be detrimental to their learning (2.09/5.00). See table 1 and figure 1.

**Table 1.** Initial Questionnaire - Number of responses

Question	Strongly Disagree (1)	Disagree (2)	Neutral (3)	Agree (4)	Strongly Agree (5)	Weighted Average	Total Responses
I anticipate that learning about the history of medicine will help me become a better doctor.	1	3	23	23	7	3.56	57
I believe that the history of medicine should be taught more in the MBBS course.	2	11	22	18	4	3.19	57
I anticipate that learning about the history of medicine will enhance my learning about pathology of diseases.	1	4	14	34	4	3.63	57
I believe that having senior medical students teach the tutorial will be beneficial to my learning.	0	4	12	26	15	3.91	57

The first questionnaire also included 2 open-ended response questions:

1. What do you think you might enjoy most about this session?
2. What do you think you will enjoy least about this session?

The first question had 28 responses and the second question had 12. All students who responded to question one mentioned that they were looking forward to the content of the tutorials. Students stated they were keen to study history as they felt it would help them place medicine into a social context, understand pathology better, and learn about the evolution of medical practice. Several students also commented they were excited to receive teaching outside of the existing curriculum.

In response to question two, students mentioned they did not want the tutorials to run overtime. Furthermore, students feared that the historical teaching would focus on dates, making lectures uninteresting and irrelevant to their learning.

### Final questionnaire

**Figure 2.**
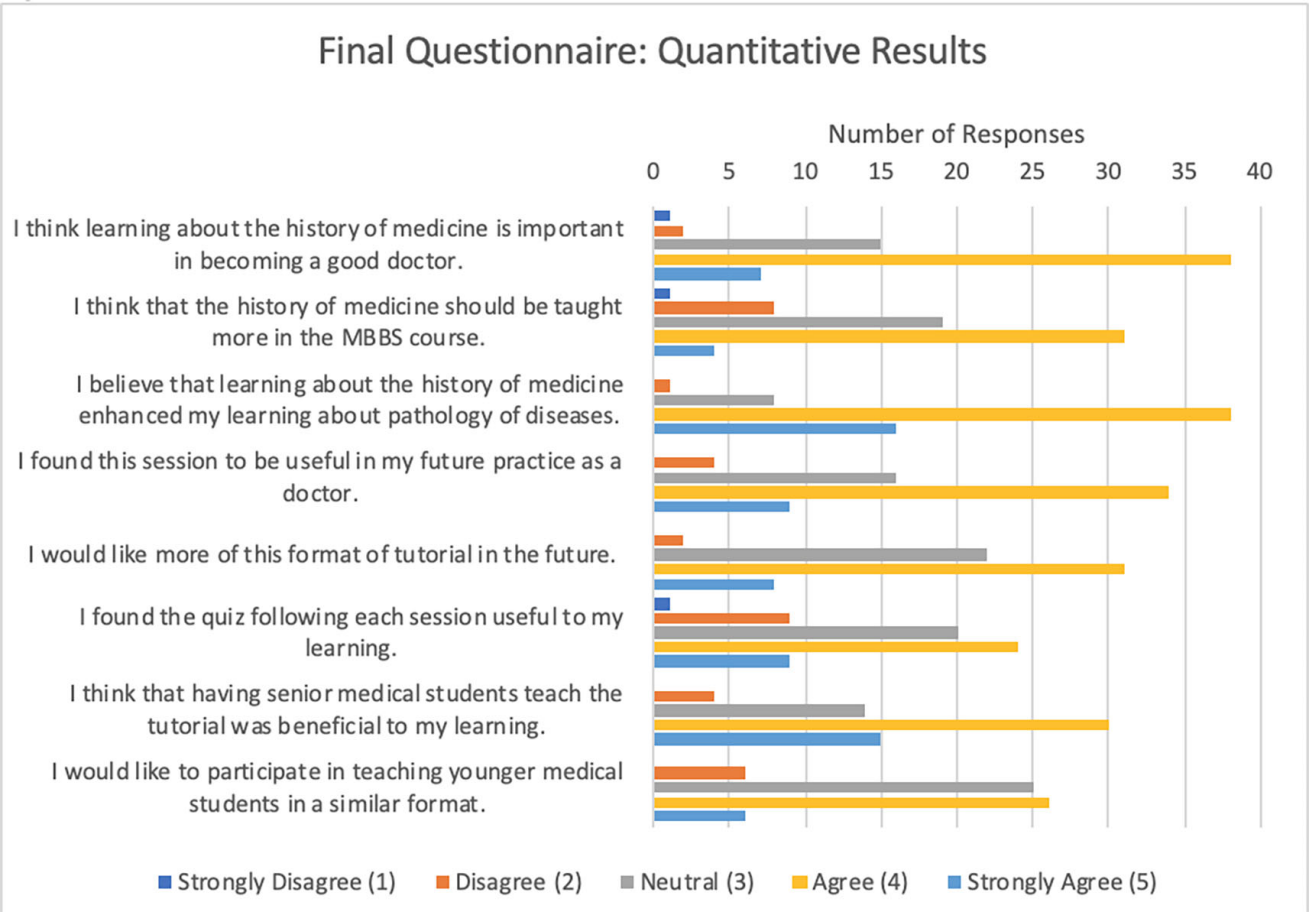
Final Questionnaire: Quantitative Results

The average response of students on the final questionnaire was that they believed learning history was important in becoming a good doctor (3.76/5.00), and that they would enjoy this lecture format in the future (3.71/5.00). Students found that integrating history with pathology was an enjoyable format of learning (4.10/5.00). They also indicated that they enjoyed the senior medical students teaching history (3.89/5.00). See table 2 and figure 2.

**Table 2.** Final Questionnaire - Number of responses

Question	Strongly Disagree (1)	Disagree (2)	Neutral (3)	Agree (4)	Strongly Agree (5)	Weighed Average	Total Responses
I think learning about the history of medicine is important in becoming a good doctor.	1	2	15	38	7	3.76	63
I think that history of medicine should be taught more in the MBBS course.	1	8	19	31	4	3.46	63
I believe that learning about the history of medicine enhanced my learning about pathology of diseases.	0	1	8	38	16	4.10	63
I found this session to be useful in my future practice as a doctor.	0	4	16	34	9	3.76	63
I would like more of this format of tutorial in the future.	0	2	22	31	8	3.71	63
I found the quiz following each session useful to my learning.	1	9	20	24	9	3.49	63
I think that having senior medical students teach the tutorial was beneficial to my learning.	0	4	14	30	15	3.89	63
I would like to participate in teaching younger medical students in a similar format.	0	6	25	26	6	3.51	63

On the final questionnaire, two open-ended questions were asked:

1. What did you enjoy most about this session?
2. What do you think could have been improved?

Of the 45 responses to question one, 16 students appreciated the content of the lessons. A common sentiment captured in one comment was that the sessions gave “background understanding of how medicine improved and evolved.” Similarly, others found that “stories were a powerful way of learning.” 18 comments indicated the integration of pathology with history was useful, specifically in retaining pathology content. Additionally, some students found that the lectures “greatly improved appreciation for medicine and history.” Several students also mentioned that historical perspectives helped to “break-up” the two-hour pathology session. Eleven comments praised the structure of the content including positive reviews of the power point slides. Another student enjoyed the “fun and casual” historical perspectives and appreciated senior medical students who led the tutorials on history of medicine, as they were “less intimidating than clinicians”.

Question two had 28 responses, of which two students commented that they found no relevance of history on clinical practice. Seven students suggested including more historical narratives rather than dates and facts. Nine commented that the structure of tutorials could be improved - specifically that historical perspectives were too rushed. Two students stated that historical perspectives should be included for all conditions rather than only selected ones. Five critiqued the teaching styles and that the information could have been delivered in a more engaging way.

## Discussion

This study indicated that students enjoyed and valued learning medical history in this format. Students provided positive responses on both initial and final questionnaires specifically to the statements on history’s importance to becoming a better doctor, and whether more history should be included in the medical curriculum. Interestingly, students’ feedback was more positive after the tutorial series compared to before, indicating the tutorials may have helped exemplify the relevance of medical history in developing as a clinician.

Students also indicated that they enjoyed the teaching of senior medical students. One reason may be that peer educators are at a closer level of learning to students (zone of proximal scaffolding) ( Vygotsky, 1978). They possess more relatable organisation of knowledge, which can be passed on to their junior peers ( Duffin, 1995). For these reasons, studies have suggested that peer educators are more adept at helping students gain understanding in basic concepts ( Roscoe & Chi, 2007).

From the qualitative feedback, students felt that historical narratives were more beneficial to learning compared to dates and facts. This idea is supported by Graesser Hoffman & Clark (1980) who found that when compared to expository texts, narratives were read faster with readers better able to recall the material.

History is an important component of medical education. It has the ability to inspire and motivate individuals, offering insights into how medical discoveries are made. While history is inspirational, it also instills humbleness with reminders of how far medicine has come - previous treatments, once considered gold standard, now proving completely obsolete ( Shedlock, Sims & Kubilius, 2012). An example used in this project was the progression of peptic ulcer management, evolving from surgical management (e.g. vagotomies) to medical (e.g histamine antagonists, proton pump inhibitors, H pylori eradication) ( Prabhu & Shivani, 2014).

History also demonstrates the humanity in medicine, in the sense that “medicine deals with nature and destiny of humans, not of machines or animals” ( Von Engelhardt, 1999, p1). A fascinating example of this concept is the ingenuity displayed by vascular surgeon Dr. Charles Rob who used his own shirts to repair a dilated abdominal aorta ( Pories, 2015). Though clearly not standard practice, Dr Rob’s creativity helped formulate a unique solution to an imposing problem.

Integrating medical history with other subjects is not a novel idea, having been successfully implemented in medical curriculums around the world ( Duffin, 1995). The integration of history and pathology helps students appreciate the relevance of history to medicine ( Huynh, 2017). Once the relevance is clear to students, they are more motivated to study the material ( Jones et.al, 2015).

### Australian Medical Council Outcome

History of medicine has value in all medical schools, particularly in the Australian context. The Australian Medical Council has a standard by which they assess medical programs. This assessment is comprised of four graduate outcome statements: 1) science and scholarship 2) clinical practice 3) health and society and 4) professionalism and leadership ( Australian Medical Council, 2012). Though history of medicine is not once mentioned in the assessment standard, it fulfills all of these outcome statements. Students gain knowledge about history (statement 1). History helps students appreciate the evolution of patient care (statement 2), and also highlights the links between health, society, and culture (statement 3). History also reminds students of the importance of professionalism and humility, as medicine is an ever-evolving entity requiring practitioners to be life-long learners (statement 4). Given the fact that teaching medical history satisfies these four statements, “it is surprising that it [history of medicine] is not taught more often in medical school” ( Huynh, 2017, p673).

### Limitations

There were roughly 120 year-three medical students enrolled at the University of Tasmania in 2018. However, only 57 pre questionnaires and 63 post questionnaires were completed. Because only half the cohort participated, it is difficult to draw definitive conclusions of what the entire cohort thought of the historical perspectives. It is likely that participation was limited by the fact that the content of the two hour session was not included in summative assessment.

Another limitation was some students provided qualitative feedback on the pathology portion of the lectures rather than the historical aspects. This confusion led to some irrelevant comments and may have caused errors in completing the quantitative portion of the questionnaire.

## Conclusion

Through this study, both research questions are answered in the affirmative. Teaching of history content was delivered successfully within an existing curriculum. Furthermore, results of the questionnaires demonstrated that medical students were interested and engaged with the course content.

There are several barriers to including history of medicine in medical curriculums. Most commonly, universities would state that their curriculums are already saturated with other content which are necessary for accreditation. However, as shown by this project, integrating existing lectures with the history of medicine may negotiate this impasse. This would infiltrate the curriculum without creating a new lecture series. Additionally, integration would help students appreciate the relevance of the history of medicine, as the past can be juxtaposed with current practice and future projections.

Overall, incorporating history of medicine into the curriculum has many benefits. This project exemplifies a practical approach to undertake the task of teaching history of medicine to medical students.

## Take Home Messages

• History of medicine is an important component of developing wholistic doctors.
• Medical history can be successfully integrated into an existing medical curriculum, ameliorating the need for creating an entirely new lecture series.
• Integrating history with patholgy helps students realise the relevance of medical history.
• Peer educators are an effective means by which to deliver course content, with the added benefit of providing a unique experience to the educators.

## Notes On Contributors

Joshua Teo is a junior doctor working in Brisbane, Australia, currently pursuing a career in general surgery. He graduated from the University of Tasmania in 2018. ORCID iD: https://orcid.org/0000-0002-7584-3286.

Aram Cox is a junior doctor working in Hobart, Australia. He is currently pursuing a career in neurosurgery. He graduated from the University of Tasmania in 2018.

Huan Doan is a junior doctor currently practicing in Newcastle, Australia. He is interested in the field of urology. He graduated from the University of Tasmania in 2018.

Tiffany Ngu is a junior doctor currently practicing in Brisbane, Australia. She is currently pursuing a career in rehabilitation medicine. She graduated from the University of Tasmania in 2017.

Professor Sinha has an extensive background in medical education and received the Medal of the Order of Australia for his outstanding contributions to medical education and wound care in 2005. He was a general surgeon practicing in Australia, India, Zambia and Papua New Guinea. He is currently a Professor in Surgery at the University of Notre Dame (Darlinghurst) and the University of Tasmania. Professor Sinha - ORCID iD: https://orcid.org/0000-0002-9676-4480
