# Supplementary material for: Infiltrating the Curriculum: Integrating Medical History with Existing Surgical Pathology Tutorials
Source: MedEdPublish (2016). 2020 Mar 12;9:44. [Version 1] doi: 10.15694/mep.2020.000044.1 (PMC10697490; doi:10.15694/mep.2020.000044.1)
Supplement: Peer review history

# Reviewer report: Reviewer response for version 1

**DOI:** 10.21956/mep.20001.r30473

**Published:** 2020-03-20

**Authors:** P Ravi Shankar

**Affiliations:** 1 American International Medical University

This review has been migrated. The reviewer awarded 4 stars out of 5 This is an interesting and well-written manuscript about ‘infiltrating’ the history of medicine during surgical pathology sessions in a medical school in Australia. The authors have described the study well and I am happy to note that final year students were involved in teaching about the history of medicine. Some of them who are now postgraduates are also the authors of the manuscript. I would also be interested in knowing more details about the topics addressed and how the sessions on the history of medicine were incorporated during the pathology sessions. How were the final year students trained to conduct the sessions?History of medicine is not a part of the curriculum in most medical schools and many faculty members still continue to question the utility of the topic. The present study can add to the increasing body of evidence highlighting the importance of the history of medicine and creating a case for their formal inclusion in the curriculum.

Reviewer Expertise:

NA

No decision status is available

# Reviewer report: Reviewer response for version 1

**DOI:** 10.21956/mep.20001.r30471

**Published:** 2020-03-15

**Authors:** Irine Sakhelashvili

**Affiliations:** 1 European University

This review has been migrated. The reviewer awarded 5 stars out of 5 It was interesting to know that medical history is not a part of the medical program in Australia. As far as I know, the curriculum of most European medical schools includes a history of medicine, at least as an elective course. Of course, it is very important to evaluate the opinion of students regarding the curriculum and the subject studied.This is all the more true when we talk about a subject that at first glance does not seem useful to the future doctor. Students, especially beginners, do not always understand why they “spend” time on "unnecessary" subjects, such as history or research skills, e.g. if it is better to study anatomy or surgery instead...As for me, the presented survey has two meanings: first, the results show that students understand and appreciate importance of historical perspectives for becoming professionals; indeed, the historical knowledge broadens our horizons; the lessons learned from experience helps us to develop; at last, this is the best way to remember and pay tribute to those who have made a huge contribution to development of medicine. The second significance of this survey is that the authors obtained significant evidence confirming the need to integrate the medical history into the educational process. Another study with similar results will be another argument for the Australian Medical Council to revise the decision to integrate medical history into the curriculum. I strongly urge that authors continue both surveys and teaching history of medicine to students. Thanks for the article.

Reviewer Expertise:

NA

No decision status is available

# Reviewer report: Reviewer response for version 1

**DOI:** 10.21956/mep.20001.r30472

**Published:** 2020-03-12

**Authors:** Balakrishnan Nair

**Affiliations:** 1 Centre for Medical Professional Development and University of Newcastle

This review has been migrated. The reviewer awarded 5 stars out of 5 This is a very interesting study about teaching history of Medicine in context. The authors should be congratulated for doing this . When we know that vaccination eradicated small pox (Jenner), antiseptics reduced mortality in surgery(Lister) and hand washing reduced post- partum sepsis ( Semmelweiss ) , we realise how far we have come. History repeats itself too ! This project highlights learning in context and peer to near teaching. I enjoyed reading this and we need more studies like this , where students and junior faculty are involved in research I wonder whether they should change the title from “ medical history “ to “history of Medicine “

Reviewer Expertise:

NA

No decision status is available
